# Eating Habits and Lifestyle during COVID-19 Lockdown in the United Arab Emirates: A Cross-Sectional Study

**DOI:** 10.3390/nu12113314

**Published:** 2020-10-29

**Authors:** Leila Cheikh Ismail, Tareq M. Osaili, Maysm N. Mohamad, Amina Al Marzouqi, Amjad H. Jarrar, Dima O. Abu Jamous, Emmanuella Magriplis, Habiba I. Ali, Haleama Al Sabbah, Hayder Hasan, Latifa M. R. AlMarzooqi, Lily Stojanovska, Mona Hashim, Reyad R. Shaker Obaid, Sheima T. Saleh, Ayesha S. Al Dhaheri

**Affiliations:** 1 Department of Clinical Nutrition and Dietetics, College of Health Sciences, University of Sharjah, Sharjah 27272, UAE; tosaili@sharjah.ac.ae (T.M.O.); haidarah@sharjah.ac.ae (H.H.); mhashim@sharjah.ac.ae (M.H.); robaid@sharjah.ac.ae (R.R.S.O.); U14120207@sharjah.ac.ae (S.T.S.); 2 Nuffield Department of Women’s & Reproductive Health, University of Oxford, Oxford OX1 2JD, UK; 3 Research Institute of Medical and Health Sciences (RIMHS), University of Sharjah, Sharjah 27272, UAE; d_abujamous@yahoo.com; 4 Department of Nutrition and Food Technology, Faculty of Agriculture, Jordan University of Science and Technology, Irbid 22110, Jordan; 5 Department of Nutrition and Health, College of Medicine and Health Sciences, United Arab Emirates University, Al Ain 15551, UAE; drmaysm@gmail.com (M.N.M.); amjadj@uaeu.ac.ae (A.H.J.); habAli@uaeu.ac.ae (H.I.A.); lily.stojanovska@uaeu.ac.ae (L.S.); ayesha_aldhaheri@uaeu.ac.ae (A.S.A.D.); 6 Department of Health Services Administration, College of Health Sciences, University of Sharjah, Sharjah 27272, UAE; amalmarzouqi@sharjah.ac.ae; 7 Department of Food Science and Human Nutrition, Agricultural University of Athens, Iera Odos 75, 11855 Athens, Greece; emagriplis@aua.gr; 8 College of Natural and Health Sciences, Zayed University, Dubai 19282, UAE; haleemah.alsabah@zu.ac.ae; 9 Nutrition Section, Ministry of Health and Prevention, Dubai 1853, UAE; latefa.rashed@moh.gov.ae; 10 Institute for Health and Sport, Victoria University, Melbourne 14428, Australia

**Keywords:** United Arab Emirates, COVID-19, eating habits, lifestyle behaviors

## Abstract

The coronavirus disease is still spreading in the United Arab Emirates (UAE) with subsequent lockdowns and social distancing measures being enforced by the government. The purpose of this study was to assess the effect of the lockdown on eating habits and lifestyle behaviors among residents of the UAE. A cross-sectional study among adults in the UAE was conducted using an online questionnaire between April and May 2020. A total of 1012 subjects participated in the study. During the pandemic, 31% reported weight gain and 72.2% had less than eight cups of water per day. Furthermore, the dietary habits of the participants were distanced from the Mediterranean diet principles and closer to “unhealthy” dietary patterns. Moreover, 38.5% did not engage in physical activity and 36.2% spent over five hours per day on screens for entertainment. A significantly higher percentage of participants reported physical exhaustion, emotional exhaustion, irritability, and tension “all the time” during the pandemic compared to before the pandemic (*p* < 0.001). Sleep disturbances were prevalent among 60.8% of the participants during the pandemic. Although lockdowns are an important safety measure to protect public health, results indicate that they might cause a variety of lifestyle changes, physical inactivity, and psychological problems among adults in the UAE.

## 1. Introduction

The novel coronavirus disease (COVID-19) pandemic has added various challenges and changes to human life worldwide, causing an unprecedented impact on human health, lifestyle, and social life, and has affected the local and international economy [1]. Following its first emergence in December 2019, in the city of Wuhan in China and its subsequent outbreak throughout the world in the following months it was characterized as a global pandemic by the World Health Organization (WHO) on 11 March 2020 [2]. On 28 September 2020, over 32.7 million confirmed cases of novel coronavirus and around 991,000 deaths worldwide were reported by the WHO [3]. In the United Arab Emirates (UAE) a total of 90,618 confirmed cases were reported in the same period [3]. In response to the rapid spread of the disease governments all around the world had to implement strict measures such as complete or partial lockdowns, isolation, quarantine and social distancing [4,5].

In the UAE, as a response to this outbreak, the government had to act quickly to contain the spread of the virus. Parallel with measures taken by most countries worldwide, complete and partial lockdowns were implemented, non-essential public places were closed, telework and distance learning was initiated, delivery services like delivering drugs to chronically ill patients were provided and sanitizing cities during night as part of the National Disinfection Program was implemented [6]. According to the World Bank, the total population of the UAE in 2019 was about 9.8 million [7]. However, nearly 75% of the population is concentrated in Abu Dhabi and Dubai as they have more than 3 million residents each. Moreover, the UAE is a multicultural country with expatriates and immigrants accounting for about 88% of the population [8]. Thus, this study provides unique opportunities to examine the impact of COVID-19 on lifestyle behaviors in the UAE.

There is no doubt that during times of confinement, food accessibility and availability may be affected, which in turn affects diet quality [9]. The imposed possibility of reduced income, job losses and anxiety about an uncertain future might lead the population to cut down expenditure including their expenses for food, making them go for more palatable, affordable and possibly unhealthy options [10]. Diet can affect many areas, but most importantly it can affect immune status [11] in the short term, a time during which heightened activity should be at its best. Available literature, however, has shown trends toward unfavorable dietary behaviors during the lockdown such as increased caloric intake, more frequent snacking, reduced consumption of fresh fruits and vegetable, and weight gain [10,12]. Traditionally, the diet in the UAE consists of fruits (such as dates), vegetables and fish and it is characterized by a high-fiber content and low fat and cholesterol content [13]; foods that characterize the Mediterranean diet and that are rich in vitamins A, D, C, folate, E and B-complex, required for an optimal immune response. Moreover, a large portion of UAE residents are from Arab countries in which fruits, vegetables and olive oils constitute key components of their diets. Therefore, it would be of interest to assess any shift in dietary habits during the COVID-19 situation.

Levels of physical activity were also negatively affected during quarantine [10,14,15]. Factors like complete lockdowns, closure of sport facilities and parks, and overall movement restrictions have reduced the ability to engage in physical activity. This was accompanied with an increase in sedentary behaviors related to quarantine, including distance learning and telework [16]. A meta-analysis on physical activity prior to COVID-19 pandemic revealed that a quarter of the population residing in the UAE had a sedentary lifestyle and were not engaged in any type of physical activity [17].

The emergence of infectious diseases reaching pandemic levels induces a huge psychological impact and distressed mental health symptoms in the population with anxiety being the most common as was shown following the Middle East respiratory syndrome coronavirus (MERS-CoV), severe acute respiratory syndrome coronavirus (SARS-CoV), and severe acute respiratory syndrome coronavirus 2 (SARS-CoV-2) [18,19]. Anxiety and uncertainty along with food insecurity and restricted healthcare access might also impact individuals with eating disorders and obesity [20,21]. Multiple factors influence the extent of psychological impact of outbreaks including unknown means of virus transmission, future unpredictability, media misinformation, and quarantine [19,22]. Consequently, such stressful events strongly aggravate disturbed sleep patterns and insomnia, poor eating habits along with decreased levels of physical activity and increased sedentary behaviors [23,24].

This study aimed to investigate the effect of quarantine on eating habits, physical activity, stress and sleep behaviors among adult UAE residents using a formulated online survey. A comparison of lifestyle and dietary behaviors before and during the lockdown was also conducted to allow better understanding of the effects of Covid-19-induced confinement policies on lifestyle changes among the UAE residents. Dietary intake was examined during the lockdown to evaluate potential risks of nutritional inadequacies.

## 2. Materials and Methods

### 2.1. Study Design and Participants

To assess the effect of the coronavirus pandemic and the effect of lockdown on eating habits and lifestyle of residents of the UAE, a population-based (cross-sectional) study was conducted in the UAE between April and May 2020. Although cross-sectional studies are rarely used to compare before and after, since there is no temporal sequence, it is the best design to use when previous information is not available, in order to draw inferences. Considering the sudden outbreak of COVID-19, this study aimed to evaluate the effect of the pandemic by examining highly modifiable factors including lifestyle and dietary.

The target population included all adults ≥18 years and from all seven emirates, residing in UAE. These were invited to participate in an online survey using snowball sampling methods in order to guarantee a large-scale distribution and recruitment of participants. A total of 1012 participants (24.1% males) were included in this study.

A web link was retrieved for the survey and was distributed using e-mail invitations and social media platforms, e.g., LinkedIn™ (Mountain View, CA, USA), Facebook™ (Cambridge, MA, USA), and WhatsApp™ (Menlo Park, CA, USA). The first page of the survey included an information sheet and consent form indicating the participants’ right to withdraw at any time. Consenting participants then chose their desired language and proceeded to complete and submit their responses. All data were collected anonymously with no indication of any personal information and participants were not rewarded. The study protocol was approved by the Research Ethics Committee at the University of Sharjah (REC-20-04-25-02) and the Social Sciences Research Ethics Committee at United Arab of Emirates University (ERS_2020_6106).

### 2.2. Survey Questionnaire

A multicomponent, self-administrated online survey was designed using Google document forms in English, Arabic, and French. This survey contained questions on dietary and lifestyle habits prior to and during the COVID-19 confinement. A researcher from the College of Health Sciences at the University of Sharjah (UAE) and a researcher from the College of Food and Agriculture at United Arab Emirates University (UAE) developed the draft of the survey in English. Questions were developed based on a previous national nutrition survey [25], the International Physical Activity Questionnaire Short Form (IPAQ-SF) [26] and the Copenhagen Psychosocial Questionnaire (COPSOQ-II) [27]. It was then translated and culturally adapted following an internationally accepted methodology [28,29]. The survey was later reviewed by the research team and was pilot tested with 25 people from the UAE. Following the pilot-testing, slight modifications were made to the survey. The online survey included 37 questions and was divided into seven sections: (1) socio-demographic background (10 questions): gender, age, marital status, number of children the participant has, education level, employment status, whether they were working or studying from home during the lockdown, weight change, perceived health status, and emirate of residence; (2) sources of information (2 questions): where do they obtain health and nutrition related information; (3) eating habits (8 questions): meal type, meal frequency, eating breakfast, skipping meals, reasons for skipping meals, water intake, and food frequency of specific foods; (4) shopping habits (5 questions): preparing a grocery list, stocking up on foods, using online shopping, reading food labels, and cleaning/sanitizing groceries; (5) physical activity (4 questions): exercising frequency, household chores frequency, computer time for work or study, and screen time for entertainment; (6) stress and irritability (4 questions): physical exhaustion, emotional exhaustion, irritability, and tension; (7) sleep (4 questions) sleep duration, sleep quality, sleep disturbances, and energy level. The full version of the questionnaire is available as a Supplementary File.

Questions on eating habits, physical activity, stress and irritability, and sleep were asked twice, once regarding the period before the pandemic (pre-COVID-19) and the other regarding the period during lockdown (during COVID-19).

#### 2.2.1. Dietary Assessment

A total of 10 specific dietary questions were included in the questionnaire to assess frequency of specific food groups only during COVID-19 pandemic [30]. Food groups were included based on usual intakes of the population residing in the United Arab Emirates [31,32]. These characterize the basic Mediterranean type diet but also include food high in sugar and fat, observed to be recently trending in the UAE [25]. Specifically, the questionnaire included the following food groups: fruit, vegetables, milk and milk products, meat and meat products (red meat, chicken and fish), grains (bread, rice pasta), sweets, sugar sweetened beverages (ssbs), coffee and tea, and energy drinks. Response options included never; 1–4 times per week; once a day; 2–3 times a day; 4 or more times a day. Internal consistency of the food added in the food frequency questionnaire was evaluated using Cronbach’s alpha for this section of the questionnaire specifically, to decrease false high internal consistency, since this test is affected by the length of the test [33]. A value of 0.81 was derived showing strong inter-relatedness of the food items, ensuring validity (Cronbach’s alpha = 0.81, from a scale of 0 to 1.0; small cohort error variance of 0.34).

#### 2.2.2. Physical Activity Assessment

A modified version of the International Physical Activity Questionnaire Short Form (IPAQ-SF) was used to assess frequency of physical activity pre-COVID-19 and during COVID-19 among surveyed participants [26]. Participants were asked to indicate “how many days per week did they engage in moderate to vigorous physical activity”, and “how many days per week did they engage in household chores”. They were also asked to indicate “how many hours per day did they spend on the computer for work or study”, and “how many hours per day did they spend on screens for fun and entertainment”.

#### 2.2.3. Stress, Irritability and Sleep Assessment

Questions on stress and sleep were adopted from the second version of the Copenhagen Psychosocial Questionnaire (COPSOQ-II) with modifications [27]. Regarding stress and irritability, participants were asked to provide the frequency of experiencing physical exhaustion; emotional exhaustion; irritability; and tension. The same questions were asked once regarding the period before the pandemic (pre-COVID-19) and once during the pandemic. The response options included all the time; a small part of the time; part of the time; a large part of the time; all the time.

With regard to sleep, participants were asked if they experienced sleep disturbances including sleeping badly and restlessly; having difficulty to go to sleep; waking up too early and not being able to get back to sleep; waking up several times and found it difficult to get back to sleep; or none of the options. The questionnaire also included the following questions: “number of sleeping hours per night”, “rating sleep quality”, and “describing energy level during the day”. The repose options for rating sleep quality were very good; good; poor. The repose options for describing energy level were energized; neutral; lazy. Questions were repeated twice, once about the period pre-COVID-19 and the second regarding the period during COVID-19.

### 2.3. Statistical Analysis

Categorical variables are presented as counts and percentages. The chi square test was used to determine the association between categorical variables, and the McNemar test was used to investigate the difference between categorical variables before and during the COVID-19 pandemic. A sub-analysis was also performed for weight and specific behavioral variables’ differences between groups. Specifically, data were stratified (i) by sex, (ii) by age group (18–35 and ≥36 years), and (iii) level of education. Principal component analysis (PCA) was used to group related dietary practice into components [34]. The correlation of each food group with the underlying component was calculated with component loadings. In this analysis, values >0.3 were considered as having an effect in the component construction. Each participant was given a score based on the sum of the component loadings of each food group. The identified components were rotated (varimax rotation) to retrieve orthogonal, uncorrelated factors, decreasing variance errors. The Kaiser–Meyer–Olkin (KMO) measure of sample adequacy was used to assess PCA adequacy. Results were significant for *p* value < 0.05. Statistical analysis was performed using Statistical Package for the Social Sciences (SPSS) version 26.0 (IBM, Chicago, IL, USA).

## 3. Results

### 3.1. Demographic Characteristics

The survey was completed by 1012 participants. The sample distribution from different emirates was representative of the population distribution in the UAE. With the highest number of participants residing in Abu Dhabi and Dubai. More specifically, local coverage spreads over all regions in the UAE: 33.9% of participants live in the capital Abu Dhabi, 32.5% in Dubai, and 33.6% in Sharjah and northern Emirates. The majority of the participants completed the survey in Arabic (60.4%), followed by English (39.3%), and only 0.3% chose the French language. Comprehensive information relating to demographic characteristics of the study population is presented in Table 1. The majority of participants were females (75.9%), aged 26–35 years (29.1%), were married (56.4%), had no children (50%), completed a bachelor’s degree (54.1%), worked full-time (53.3%), and were working or studying from home during quarantine (61.6%). Almost one third of the participants reported weight gain since the start of the lockdown (31%). However, 20.9% reported weight loss, 40.1% maintained their weight, and 7.9% did not know if there was a change in their weight. The majority of participants described their health status during the outbreak as very good (39.7%) and only 0.7% indicated poor health status.

### 3.2. Source of Information

When asked about the most common source of information for health and nutrition updates, 69.1% and 67.8% of participants reported relying on social media applications, respectively (Table 2). Local and international health authorities were selected as the second source of information for both health and nutrition updates (65.4% and 48.7%, respectively).

### 3.3. Eating Habits

Table 3 presents the eating habits of the study participants pre- and during the COVID-19 pandemic. Results showed a significant increase in the percentage of participants consuming mostly homemade meals during the pandemic and a significant reduction in those mainly consuming fast-food (*p* < 0.001). Moreover, the percentage of participants consuming five or more meals per day increased from 2.1% before the pandemic to 7% during the pandemic (*p* < 0.001). Also, the percentage of participants consuming breakfast increased from 66% to 74.2%, and the percentage of those skipping meals decreased from 64.5% to 46.2% during the pandemic (*p* < 0.001). Participants reported skipping meals mainly due to lack of time before the pandemic (62.3%), however, the main reason behind that was lack of appetite (36%). With regards to water intake, only 24.1% of participants consumed eight or more cups per day before the pandemic, and the percentage increased to 27.8% during the pandemic (*p* = 0.003).

The frequency of consumption for particular food products during the COVID-19 pandemic among residents of the UAE are presented in Table 4. Over half of the participants (51.2%) did not consume fruits daily, 37% did not consume vegetables daily, and 46.2% did not consume milk and dairy products on daily basis. However, 46.1% of the participants consumed sweets and desserts at least once per day, and 37.1% reported consuming salty snacks (chips, crackers, and nuts) every day.

Additionally, 69.2% had tea or coffee at least once per day. Sweet drinks such as fruit juices and beverages were less popular among the study participants, as 44.2% reported never consuming them and an even higher percentage (86.5%) reported never consuming energy drinks during the pandemic.

A total of two components from the PCA output were derived, based on eigenvalue (at least 1) and scree plots obtained (Table 5). These two components explained 47% of the variance in eating behavior and were named based on the interpretation of the component loadings. The first pattern explained 31% of eating variation and was named “Western-type diet” since it was characterized by significantly positive loadings in dairy, meat, sweets, salted foods and vegetables. The second pattern explained 16% of the variance and loaded positively with ssbs and energy drinks and negatively on fruits and vegetables. Therefore, it was named “Free Sugars diet”. A KMO of 0.78 was obtained, which is considered substantial.

### 3.4. Shopping

The results revealed that the majority of participants prepared a shopping list beforehand (80.3%), started stocking up on foods during the pandemic (43.9%), did not order their groceries online (58.0%), read the food label before purchasing products (52.4%), and sanitized or cleaned groceries before storing them (71.9%) (Table 6).

### 3.5. Physical Activity

**Figure 1:**
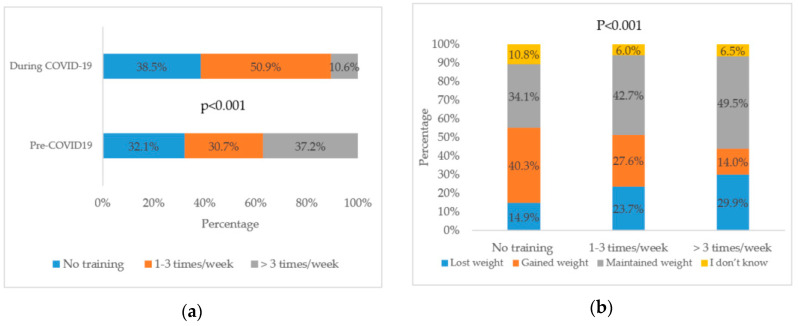
Physical activity pre- and during COVID-19 pandemic (**a**) Frequency; (**b**) Change in weight. The *p* values indicate the statistical significance of McNemar test. The *p* values indicate the statistical significance of chi-square test.

Figure 1a shows that 32.1% of the participants reported not engaging in any physical activity before the coronavirus pandemic, and the percentage increased to 38.5% during the pandemic (*p* < 0.001). Moreover, Figure 1b shows that there was a significant association between the frequency of performing physical activity during the pandemic and the reported change in weight among participants (*p* < 0.001). Of those who reported performing physical activity more than three times per week, 29.9% lost weight and 49.5% maintained their weight (*p* < 0.001). Furthermore, 40.3% of people who did not perform physical activity reported weight gain.

A significantly higher percentage of participants spent more than five hours per day on the computer for study or work purposes during the pandemic (47.6%) compared to before the pandemic (32%) (*p* < 0.001). Similarly, the percentage of participants spending more than five hours per day on screens for fun increased from 12.9% before the lockdown to 36.2% during the lockdown (*p* < 0.001) (Table 7).

### 3.6. Stress

**Figure 2:**
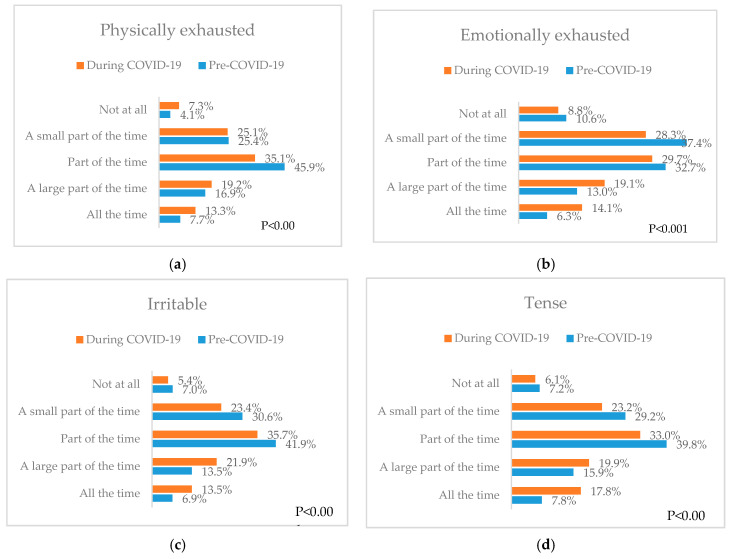
Stress and irritability pre- and during COVID-19 pandemic (**a**) Physical exhaustion; (**b**) Emotional exhaustion; (**c**) Irritability; (**d**) Tension. The *p* values indicate the statistical significance of McNemar test.

Participants were asked to indicate the frequency of experiencing physical exhaustion; emotional exhaustion; irritability; and tension before and during the pandemic. Figure 2 presented the response distribution in percentages for each of the four stress parameters.

The results indicate a significant increase in the percentage of participants reporting all four stress parameters “all the time” during the coronavirus pandemic compared to before the pandemic (13.3% vs. 7.7% for physical exhaustion; 14.1% vs. 6.3% for emotional exhaustion; 13.5% vs. 6.9% for irritability; and 17.8% vs. 6.3% for tension) (all *p* < 0.001).

### 3.7. Sleep

Results showed a significant decrease in the percentage of participants who reported sleeping less than seven hours per night from 51.7% before the pandemic to 39% during the pandemic (*p* < 0.001) (Table 8). However, a higher percentage of participants reported poor sleep quality during the pandemic (28.1%) compared to before the pandemic (17.3) (*p* < 0.001), and sleep disturbances were also more common during the pandemic (60.8%) compared to before (52.9%). Consequently, 30.9% of the surveyed participants reported feeling lazy and less energized during the pandemic, compared to only 4.7% before the pandemic (*p* < 0.001) (Table 8).

An analysis of weight and behavioral factors by sex and age groups is depicted in Table 9. Significantly more males reported decreased engagement in physical activity (50% vs. 39.3%; *p* = 0.013) and increased screen time (54.5% vs. 51%; *p* = 0.002). Sleep disturbances increase was, however, significantly higher in females (*p* = 0.011). Moreover, those aged over 36 years reported a higher weight gain as well as an increase in the number of meals consumed per day (*p* = 0.042 and *p* = 0.024, respectively). Sleep duration and quality was most affected among participants aged 18–35 (*p* < 0.001). There was no significant association between different education levels and lifestyle changes (Table 9).

## 4. Discussion

This population-based, cross-sectional study assessed eating habits and lifestyle behaviors among residences of the UAE, via an online survey during the COVID-19 pandemic between April and May 2020. The results indicate that the COVID-19 pandemic and the subsequent lockdown resulted in weight gain in about one-third of the respondents with changes in important and highly modifiable dietary and lifestyle behaviors that are considered essential for optimal somatic and psychological health. Specifically, participants also reported an increase in the number of meals consumed per day and a reduction in the percentage of skipping meals particularly breakfast during the pandemic. The present study also indicated that dietary habits were distanced from the Mediterranean diet principles and closer to “unhealthy” dietary patterns, characterized as high in energy but with low nutrient density; viewed as a detrimental combination for immune status. Although more homemade meals were prepared, a factor associated with healthy weight status, at the same time more non-nutritious foods were chosen, as well as being more frequently consumed (since an increase was also seen among frequency of meals per day). These data, therefore, are informative on the potential alterations of food prepared and consumed although at home.

In agreement with our study, the results from Kuwait, United States, Italy and France revealed an increase in caloric intake and indicated weight gain during the current COVID-19 home confinement [10,35,36,37]. Data from Kuwait, a close Gulf country to UAE, showed a significant increase in weight of respondents during the quarantine and the weight gain was 4.5 times higher among those consuming unhealthy diets [38]. The actual weight increase was not assessed in this study considering the short time interval of COVID-19 lockdown, however, the large percentage of the population that reported an increase in weight can be used as a proxy pertaining to changes in eating behavior and activity level. It has been suggested that the negative alterations in eating behaviors could be due to anxiety or boredom [39], lack of motivation to maintain healthy habits [40], or reduced availability of goods and limited access to food due to restricted store opening hours [41]. The prevalence of overweight and obesity in the UAE even before COVID-19 was high and has increased over time [42]. It is estimated that over one third of the population in the UAE is living with obesity with higher rates among females [43]. Thus, extra efforts are needed to reduce the burden of obesity and its risk factors especially during the COVID-19 pandemic.

Over half of the surveyed participants in this study did not consume fruits daily and about one third did not consume vegetables and dairy products on daily basis. Instead, almost half of the same population reported consuming sweets and desserts at least once per day and over one third consumed salty snacks daily. This transition towards a Westernized diet in the UAE was reported in 1998, where the consumption of fresh fruit and vegetables and of milk and dairy products was found low [32]. Moreover, in 2003, 77.5% of males and 75.7% of females in the UAE had less than five servings of fruit and vegetables per day [44]. Likewise, a recent study among Emirati adolescents revealed that only 28% of them met the recommended daily fruit and vegetable intake [45]. This is concerning especially as fruits and vegetables are an important source of fiber, vitamins, minerals, and antioxidants. Diets rich in antioxidants (such as the Mediterranean diet and Dietary Approaches to Stop Hypertension (DASH) diet) are vascular protective. The Mediterranean diet is recognized as an anti-inflammatory dietary pattern, focusing on high consumption of plant foods, low red meat and dairy and moderate consumption of monounsaturated fat sources such as olive oil [46]. Evidence suggests that the Mediterranean diet is associated with better health status, lower risk of chronic disease and inflammation as well as increased immunity [47,48,49]. The Mediterranean diet is not only a healthy dietary pattern, but is also a sustainable diet that has a lower environmental impact than the typical Western diet [50]. Moreover, mounting evidence indicates that the Mediterranean diet has a favorable effect on diseases related to chronic inflammation, including visceral obesity, type 2 diabetes mellitus and the metabolic syndrome [51,52,53,54,55]. Knowing that the prevalence of cardiovascular disease incidence is high in the UAE (40%) [56] and rates of dyslipidemia are strikingly elevated (72.5%) [57] makes it imperative that diets such as the Mediterranean diet should be encouraged to prevent the potentially negative effect of quarantine on dietary habits and overall health [41].

Due to the increase in obesogenic behaviors related to the COVID-19 pandemic, two dietary patterns were revealed among the studied population, named the “Western-type diet” and the “Free Sugars diet”. These patterns indicate unhealthy eating behaviors during the period of the pandemic. This is in agreement with previous studies reporting a transformation of the diet in Eastern Mediterranean countries from a traditional Mediterranean diet to a more Westernized diet which is high in energy, saturated fat, cholesterol, salt, and refined carbohydrates, and low in fruits, vegetables, fiber, and polyunsaturated fats [25,58,59,60]. Therefore, current dietary behaviors in the UAE may not be effective against the COVID-19 virus since it can adversely affect the immune system response among other health factors. Furthermore, it is unclear whether these dietary patterns were due to the lockdown that followed the COVID-19 outbreak; however, the implications can be detrimental considering an adequate supply of macro- and micro-nutrients are essential for optimal immune function and response [11,61].

Amidst these passive changes in food behavior, some beneficial aspects emerged from this study, such as a significant increase in home-made food preparations, regular breakfast consumption and lower intakes of fast foods. Similarly, a consumer online based survey conducted by Ipsos across the Middle East and North Africa (MENA) region revealed that 57% out of the 5000 consumers who took part in the survey were preparing their own meals, and 79% were eating less often at restaurants [62].

Among the surveyed participants, more than one third reported a non-engagement in any physical activity during coronavirus pandemic lockdown. This was mostly observed among males in this study, with a simultaneously greater likelihood of increased sedentary time, compared to females. The findings of this questionnaire are in accordance with other studies indicating that the current COVID-19 pandemic had a dramatic impact on lifestyle behaviors globally, including diminished engagement in sports and physical activity in general [63,64,65]. Moreover, the “Effects of home Confinement on multiple Lifestyle Behaviours during the COVID-19 outbreak (ECLB-COVID-19)” international survey revealed that the COVID-19 pandemic had a negative effect on all levels of physical activity (vigorous, moderate, walking and overall) and increased daily sedentary time by more than 28% [14]. Similarly, in the current study the proportion of participants who spent more than five hours per day on screens for entertainment increased by 23.3%. Together with the unhealthy diet, the reduction of physical activity would not only contribute to weight gain, but also to an increase in cardiovascular risk during quarantine. Thus, awareness about the importance of regular physical activity and its benefits on overall health is necessary during such times [66,67]. It is also important to identify groups at a higher risk of unhealthy lifestyle behaviors during the COVID-19 pandemic to design interventions targeted towards these groups.

During the COVID-19 pandemic higher levels of anxiety, stress and depression have been observed among individuals [68,69,70]. In this study, the percentage of participants experiencing exhaustion, irritability, and tension more often during the coronavirus pandemic increased significantly. Sleep was mostly affected in females and needs to be further evaluated since it is linked with multiple endocrine functions, as well risk for obesity and depression. The risk of obesity is underlined by the significant increase in daily meal frequency among participants over 36 years with the majority being female. Also, despite WHO recommendations to minimize listening to unreliable news that could cause anxiety or distress and to seek information only from trusted sources [71], over two thirds of participants in this survey used social media as a main source for health updates. Studies have shown the negative and harmful effect of misinformation overload “infodemic” on the mental health of individuals [72,73]. Moreover, stress and anxiety could disrupt sleep quality during the night and energy levels during the day. Results of the current survey indicated a 10.8% increase in participants reporting poor sleep quality and 26.2% increase in those feeling lazy during the pandemic. Xiao and his co-workers found a significant negative correlation between anxiety levels and sleep quality and suggested the use of telepsychiatry consultation as an important therapeutic strategy [74]. The use of telehealth has been shown to be useful in providing support to patients and is appropriate for the delivery of mental health services [75]. Additionally, the Mediterranean diet does not only have a protective effect on the risk of cardiovascular diseases and certain types of cancer [54,76], but also an increased compliance with it could be associated with lesser mental distress, better sleep quality, and higher scoring for self-perceived health status [77,78,79].

It is acknowledged that this study has limitations related to the use of self-reported questionnaire, snowball sampling method and the cross-sectional study design. The study information was acquired after lockdown, and although comparisons are critical to be made in order to draw inferences, no conclusive remarks can be drawn. Results stratified by sex should be interpreted with caution, since the majority of the participants were females. Furthermore, in order to minimize selection bias that may arise with snowball sampling (including interrelated-similar individuals), each individual could refer a maximum of three people who were not family members, and only one individual per age group (young adults, older adults, elderly) was enrolled from a household. Moreover, the change in dietary pattern was not assessed in this study, since data on food frequency were only obtained during COVID-19 pandemic, although these can be used as a reference for further studies performed, in these uncertain times. This was done to reduce the probability of including recall bias, since the participants had to respond to multiple questions on food frequency and quantity during COVID-19 lockdown and for a prolonged period prior to that. Also, the presence of obesity and eating disorders were not determined in the study, nor was information on infection with COVID-19 reported. Such analysis would require a longer questionnaire, hence may have decreased the compliance and response rate, but also would have required a larger sample size based on the prevalence of all factors to acquire adequate study power. Another potential limitation of the study was that respondents were mostly females. Although this is usual in online questionnaires [80], it should be considered when generalizing the results. However, using an online survey facilitated data collection during COVID-19 pandemic from all seven emirates. It also guaranteed the anonymity of the participants, thus reducing the social desirability bias. The strengths of this research include data collection timing one month after lockdown which minimizes memory failure for previous habits. In addition, the survey provided was in multiple languages in a multilingual environment like UAE.

The results of the study indicate that individuals in the UAE experienced negative lifestyle changes, unbalanced food choices, a reduction in physical activity, and psychological problems during the COVID-19 pandemic. Although quarantine is an essential measure to protect public health and control the transmission of the virus, these findings should be taken into consideration for future regulations in the UAE.

## Figures and Tables

**Table 1:** Demographic characteristics of study participants (*n* = 1012).

Characteristics	*n*	%
Gender		
Male	244	24.1
Female	768	75.9
Age (years)		
18–25	280	27.7
26–35	294	29.1
36–45	240	23.7
46–55	154	15.2
>55	44	4.3
Marital status		
Married	571	56.4
Single	403	39.8
Divorced	30	3.0
Widowed	8	0.8
Number of children		
None	506	50.0
1–2	230	22.7
≥ 3	276	27.3
Education level		
Less than high school	8	0.8
High school	111	11.0
College/Diploma	102	10.1
Bachelor’s degree	547	54.1
Higher than bachelor’s degree	244	24.1
Employment status		
Full-time	539	53.3
Part-time	44	4.3
Self-employed	31	3.1
Student	156	15.4
Unemployed	230	22.7
Retired	12	1.2
Working/studying from home		
Yes	623	61.6
No	309	30.5
Not applicable	80	7.9
Weight change during pandemic		
Lost weight	212	20.9
Gained weight	314	31.0
Maintained weight	406	40.1
Do not know	80	7.9
Perceived health state during pandemic		
Excellent	217	21.4
Very good	402	39.7
Good	284	28.1
Fair	102	10.1
Poor	7	0.7
Emirate of residence		
Abu Dhabi	343	33.9
Dubai	329	32.5
Sharjah	244	24.1
Ajman	52	5.1
Ras al Khaimah	20	2.0
Fujairah	16	1.6
Umm al Quwain	8	0.8

**Table 2:** Source of health and nutrition information during COVID-19 pandemic (*n* = 1012).

Source of Information *	Health-Related Information, *n* (%)	Nutrition-Related Information, *n* (%)
Local and international health authorities	662 (65.4)	493 (48.7)
Social media	699 (69.1)	686 (67.8)
Healthcare professionals	409 (40.4)	462 (45.7)
Television	231 (22.8)	172 (17.0)
Newspapers	75 (7.4)	51 (5.0)
Friends and family	339 (33.5)	386 (38.1)

* As multiple responses were allowed, the total number of responses is greater than the number of surveyed participants and the percent of cases is displayed.

**Table 3:** Eating habits pre- and during COVID-19 pandemic (*n* = 1012).

Variables	Pre-COVID-19 *n* (%)	During COVID-19 *n* (%)	*p*-Value (2-Sided)
Most consumed meals during the week *			
Homemade	838 (82.8)	974 (96.2)	<0.001
Frozen ready-to-eat meals	119 (11.8)	97 (9.6)	0.032
Fast food	270 (26.7)	80 (7.9)	<0.001
Restaurants ^1^	289 (28.6)	58 (5.7)	<0.001
Healthy restaurants ^2^	98 (9.7)	46 (4.5)	<0.001
Number of meals per day			
1–2 meals	470 (46.4)	369 (36.5)	<0.001
3–4 meals	521 (51.5)	572 (56.5)	0.009
≥5 meals	21 (2.1)	71 (7.0)	<0.001
Eating breakfast on most days			
Yes	668 (66.0)	751 (74.2)	<0.001
No	344 (34.0)	261 (25.8)	
Skipping meals			
Yes	663 (65.5)	468 (46.2)	<0.001
No	349 (34.5)	544 (53.8)	
Reasons for skipping meals (If the answer was yes) *			
To reduce food intake	143 (21.7)	136 (29.1)	0.011
Lack of time	410 (62.3)	143 (30.6)	<0.001
To lose weight	122 (18.5)	110 (23.6)	0.001
Lack of appetite	182 (27.7)	168 (36.0)	0.016
Fasting	68 (10.3)	120 (25.7)	<0.001
Amount of water consumed per day			
1–4 cups	410 (40.5)	337 (33.3)	<0.001
5–7 cups	358 (35.4)	394 (38.9)	0.036
≥8 cups	244 (24.1)	281 (27.8)	0.003

* As multiple responses were allowed, the total number of responses is greater than the number of surveyed participants and the percent of cases is displayed. ^1^ Restaurants: included all ethnic restaurants (Asian, Middle Eastern, International, etc.), casual dining and family style restaurants; ^2^ healthy restaurants: included food outlets with the “Weqaya logo”, restaurants categorized as “healthy” on food mobile apps (such as Zomato, Talabat, and Uber Eats) or catering services providing meal plan services based on nutritional needs (such as Kcal, right bite, Eat Clean ME, etc.).

**Table 4:** The frequency of consumption of particular foods during COVID-19 pandemic (*n* = 1012).

Food Items	≥4 Times/Day	2–3 Times/Day	Once/Day	1–4 Times/Week	Never
	*n* (%)				
Fruits	20 (2.0)	133 (13.1)	341 (33.7)	462 (45.7)	56 (5.5)
Vegetables	32 (3.2)	244 (24.1)	362 (35.8)	356 (35.2)	18 (1.8)
Milk and milk products	17 (1.7)	167 (16.5)	361 (35.7)	374 (37.0)	93 (9.2)
Meat/fish/chicken	32 (3.2)	133 (13.1)	440 (43.5)	383 (37.8)	24 (2.4)
Bread/rice/pasta	43 (4.2)	263 (26.0)	350 (34.6)	311 (30.7)	45 (4.4)
Sweets/desserts	29 (2.9)	106 (10.5)	331 (32.7)	437 (43.2)	109 (10.8)
Salty snacks	14 (1.4)	85 (8.4)	276 (27.3)	500 (49.4)	137 (13.5)
Coffee/tea	80 (7.9)	321 (31.7)	300 (29.6)	222 (21.9)	89 (8.8)
Sweetened drinks	18 (1.8)	51 (5.0)	156 (15.4)	340 (33.6)	447 (44.2)
Energy drinks	4 (0.4)	11 (1.1)	35 (3.5)	87 (8.6)	875 (86.5)

**Table 5:** Component loading for the two major dietary patterns of the participants during COVID-19.

Food Groups	Western	Free Sugars
Fruits	0.2839	**−0.3807**
Vegetable	**0.3302**	**−0.4219**
Milk	**0.3247**	−0.1932
Meat	**0.3599**	−0.0732
Carbs	**0.3975**	−0.0764
Sweets	**0.3845**	0.2917
Salted Foods	**0.3356**	0.2776
Coffee/Tea	0.2457	−0.1641
Sweet Drinks	0.2678	**0.4929**
Energy Drinks	0.1575	**0.4433**
**KMO**	**0.78**	

KMO: Kaiser–Meyer–Olkin (KMO) test. The unique characteristics of each component (dietary pattern) are presented in bold. Marginally unique dietary characteristic for each component. Loadings ≥0.30 and ≤−0.30.

**Table 6:** Shopping practices during COVID-19 pandemic (*n* = 1012).

Variables	*n*	%
Prepare shopping list		
Yes	813	80.3
No	199	19.7
Start stocking up on foods		
Yes	444	43.9
No	412	40.7
Already stocking up	156	15.4
Online grocery shopping		
Yes	425	42.0
No	587	58.0
Reading food labels		
Yes	530	52.4
No	113	11.2
Sometimes	369	36.5
Sanitizing/cleaning groceries		
Yes	728	71.9
No	113	11.2
Sometimes	171	16.9

**Table 7:** Daily activities pre- and during COVID-19 pandemic (*n* = 1012).

Variables	Pre-COVID-19 *n* (%)	During COVID-19 *n* (%)	*p*-Value (2-Sided)
Doing household chores			
Never	302 (29.8)	207 (20.5)	<0.001
1–3 times/week	404 (39.8)	333 (32.9)	<0.001
4–5 times/week	62 (6.1)	114 (11.3)	<0.001
Everyday	244 (24.1)	358 (35.4)	<0.001
Screen time for study or work			
None	188 (18.6)	160 (15.8)	0.004
1–2 h/day	282 (27.9)	136 (13.4)	<0.001
3–5 h/day	218 (21.5)	234 (23.1)	0.375
>5 h/day	324 (32.0)	482 (47.6)	<0.001
Screen time for entertainment			
Less than 30 min/day	113 (11.2)	62 (6.1)	<0.001
1–2 h/day	456 (45.1)	231 (22.8)	<0.001
3–5 h/day	312 (30.8)	353 (34.9)	0.053
>5 h/day	131 (12.9)	366 (36.2)	<0.001

**Table 8:** Sleep pre- and during COVID-19 pandemic (*n* = 1012).

Variables	Pre-COVID-19 *n* (%)	During COVID-19 *n* (%)	*p*-Value (2-Sided)
Hours of sleep per night			
<7 h	523 (51.7)	395 (39.0)	<0.001
7–9 h	459 (45.4)	499 (49.3)	0.057
>9 h	30 (3.0)	118 (11.7)	<0.001
How would you rate your sleep quality			
Very good	308 (30.4)	282 (27.9)	0.134
Good	529 (52.3)	446 (44.1)	<0.001
Poor	175 (17.3)	284 (28.1)	<0.001
Did you experience any of the following *			
Slept badly and restlessly	251 (24.8)	285 (28.2)	0.057
Hard to go to sleep	199 (19.7)	358 (35.4)	<0.001
Woken up too early and not been able to get back to sleep	232 (22.9)	147 (14.5)	<0.001
Woken up several times and found it difficult to get back to sleep	187 (18.5)	334 (33.0)	<0.001
None	477 (47.1)	397 (39.2)	<0.001
Describe your energy level			
Energized	369 (36.5)	189 (18.7)	<0.001
Neutral	596 (58.9)	510 (50.4)	<0.001
Lazy	47 (4.7)	313 (30.9)	<0.001

* As multiple responses were allowed, the total number of responses is greater than the number of surveyed participants and the percent of cases is displayed.

**Table 9:** Lifestyle changes during COVID-19 pandemic by demographic factors (*n* = 1012).

Variables	All *n* = 1012	Gender			Age Group (Year)			Education Level		
		Female *n* = 768	Male *n* = 244	*p* Value	18–35 *n* = 574	≥36 *n* = 438	*p* Value	High School *n* = 119	Higher Degree *n* = 893	*p* Value
**Weight, n, (%)**										
Decreased	212 (20.9)	166 (21.6)	46 (18.9)	0.143	131 (22.8)	81 (18.5)	0.042	19 (16.0)	193 (21.6)	0.350
Same as before	486 (48.0)	376 (49.0)	110 (45.1)		273 (47.6)	213 (48.6)		62 (52.1)	424 (47.5)	
Increased	314 (31.0)	226 (29.4)	88 (36.1)		170 (29.6)	144 (32.9)		38 (31.9)	276 (30.9)	
**Meals per day, n (%)**										
Decreased	124 (12.3)	96 (12.5)	28 (11.5)	0.140	84 (14.6)	40 (9.1)	0.024	13 (10.9)	111 (12.4)	0.352
Same as before	628 (62.1)	464 (60.4)	164 (67.2)		342 (59.6)	272 (61.9)		69 (58.0)	559 (62.6)	
Increased	260 (25.7)	208 (27.1)	52 (21.3)		148 (25.8)	127 (29.0)		37 (31.1)	223 (25.0)	
**Physical activity, n (%)**										
Decreased	424 (41.9)	302 (39.3)	122 (50.0)	0.013	226 (39.4)	198 (45.2)	0.171	42 (35.3)	382 (42.8)	0.169
Same as before	438 (43.3)	346 (45.1)	92 (37.7)		258 (44.9)	180 (41.1)		61 (51.3)	377 (42.2)	
Increased	150 (14.8)	120 (15.6)	30 (12.3)		90 (15.7)	60 (13.7)		16 (13.4)	134 (15.0)	
**Screen time (entertainment), n (%)**										
Decreased	72 (7.1)	67 (8.7)	5 (2.0)	0.002	46 (8.0)	26 (5.9)	0.150	8 (6.7)	64 (7.2)	0.984
Same as before	415 (41.0)	309 (40.2)	106 (43.4)		222 (38.7)	193 (44.1)		49 (41.2)	366 (41.0)	
Increased	525 (51.9)	392 (51.0)	133 (54.5)		306 (53.3)	219 (50.0)		62 (52.1)	463 (51.8)	
**Sleep (h), n (%)**										
Decreased	148 (14.6)	124 (16.1)	24 (9.8)	0.051	100 (17.4)	48 (11.0)	<0.001	23 (19.3)	125 (14.0)	0.302
Same as before	534 (52.8)	397 (51.7)	137 (56.1)		270 (47.0)	264 (60.3)		59 (49.6)	475 (53.2)	
Increased	330 (32.6)	247 (32.2)	83 (34.0)		204 (35.5)	126 (28.8)		37 (31.1)	293 (32.8)	
**Sleep disturbances, n (%)**										
Decreased	157 (15.5)	119 (15.5)	38 (15.6)	0.011	90 (15.7)	67 (15.3)	<0.001	16 (13.4)	141 (15.8)	0.135
Same as before	552 (54.5)	401 (52.2)	151 (61.9)		285 (49.7)	267 (61.0)		58 (48.7)	494 (55.3)	
Increased	303 (29.9)	248 (32.3)	55 (22.5)		199 (34.7)	104 (23.7)		45 (37.8)	258 (28.9)	

*p* value was based on chi-square test at 5% level.

## Supplementary Materials

The following are available online at https://www.mdpi.com/2072-6643/12/11/3314/s1, Eating Habits and Lifestyle during COVID-19 Lockdown in the United Arab Emirates: A Cross-Sectional Study.

## References

[1] Barro R.J., Ursúa J.F., Weng J. (2020). The Coronavirus and the Great Influenza Pandemic: Lessons from the “Spanish flu” for the Coronavirus’s Potential Effects on Mortality and Economic Activity.

[2] WHO WHO Director-General’s Opening Remarks at the Media Briefing on COVID-19—11 March 2020. https://www.who.int/dg/speeches/detail/who-director-general-s-opening-remarks-at-the-media-briefing-on-covid-19---11-march-2020.

[3] WHO Coronavirus Disease (COVID-19) Weekly Epidemiological and Operational Updates September 2020. https://www.who.int/docs/default-source/coronaviruse/situation-reports/20200928-weekly-epi-update.pdf?sfvrsn=9e354665_6.

[4] Wilder-Smith A., Freedman D. (2020). Isolation, quarantine, social distancing and community containment: Pivotal role for old-style public health measures in the novel coronavirus (2019-nCoV) outbreak. J. Travel Med..

[5] Koh D. (2020). COVID-19 lockdowns throughout the world. Occup. Med..

[6] Bloukh S.H., Shaikh A., Pathan H.M., Edis Z. (2020). Prevalence of COVID-19: A Look behind the Scenes from the UAE and India. Preprints.

[7] Bank T.W. United Arab Emirates: Data Source: United Nations World Population Prospects. https://data.worldbank.org/country/AE.

[8] De Bel-Air F. (2015). Demography, Migration, and the Labour Market in the UAE.

[9] Scarmozzino F., Visioli F. (2020). Covid-19 and the Subsequent Lockdown Modified Dietary Habits of Almost Half the Population in an Italian Sample. Foods.

[10] Deschasaux-Tanguy M., Druesne-Pecollo N., Esseddik Y., Szabo de Edelenyi F., Alles B., Andreeva V.A., Baudry J., Charreire H., Deschamps V., Egnell M. (2020). Diet and physical activity during the COVID-19 lockdown period (March-May 2020): Results from the French NutriNet-Sante cohort study. medRxiv.

[11] Calder P.C. (2020). Nutrition, immunity and COVID-19. BMJ Nutr. Prev. Health.

[12] Zachary Z., Brianna F., Brianna L., Garrett P., Jade W., Alyssa D., Mikayla K. (2020). Self-quarantine and Weight Gain Related Risk Factors During the COVID-19 Pandemic. Obes. Res. Clin. Pract..

[13] Musaiger A.O. (2002). Diet and Prevention of Coronary Heart Disease in the Arab Middle East Countries. Med. Princ. Pract..

[14] Ammar A., Brach M., Trabelsi K., Chtourou H., Boukhris O., Masmoudi L., Bouaziz B., Bentlage E., How D., Ahmed M. (2020). Effects of COVID-19 Home Confinement on Eating Behaviour and Physical Activity: Results of the ECLB-COVID19 International Online Survey. Nutrients.

[15] Lippi G., Henry B.M., Sanchis-Gomar F. (2020). Physical inactivity and cardiovascular disease at the time of coronavirus disease 2019 (COVID-19). Eur. J. Prev. Cardiol..

[16] Hall G., Laddu D.R., Phillips S.A., Lavie C.J., Arena R. (2020). A tale of two pandemics: How will COVID-19 and global trends in physical inactivity and sedentary behavior affect one another?. Prog. Cardiovasc. Dis..

[17] Yammine K. (2017). The prevalence of physical activity among the young population of UAE: A meta-analysis. Perspect. Public Health.

[18] Wu P., Fang Y., Guan Z., Fan B., Kong J., Yao Z., Liu X., Fuller C.J., Susser E., Lu J. (2009). The psychological impact of the SARS epidemic on hospital employees in China: Exposure, risk perception, and altruistic acceptance of risk. Can. J. Psychiatry.

[19] Pfefferbaum B., North C.S. (2020). Mental health and the Covid-19 pandemic. N. Engl. J. Med..

[20] Todisco P., Donini L.M. (2020). Eating disorders and obesity (ED&O) in the COVID-19 storm. Eat. Weight Disord..

[21] Touyz S., Lacey H., Hay P. (2020). Eating disorders in the time of COVID-19. J. Eat. Disord..

[22] Rajkumar R.P. (2020). COVID-19 and mental health: A review of the existing literature. Asian J. Psychiatry.

[23] Holmes E.A., O’Connor R.C., Perry V.H., Tracey I., Wessely S., Arseneault L., Ballard C., Christensen H., Silver R.C., Everall I. (2020). Multidisciplinary research priorities for the COVID-19 pandemic: A call for action for mental health science. Lancet Psychiatry.

[24] Torales J., O’Higgins M., Castaldelli-Maia J.M., Ventriglio A. (2020). The outbreak of COVID-19 coronavirus and its impact on global mental health. Int. J. Soc. Psychiatry.

[25] Ng S.W., Zaghloul S., Ali H., Harrison G., Yeatts K., El Sadig M., Popkin B.M. (2011). Nutrition transition in the United Arab Emirates. Eur. J. Clin. Nutr..

[26] Lee P.H., Macfarlane D.J., Lam T.H., Stewart S.M. (2011). Validity of the international physical activity questionnaire short form (IPAQ-SF): A systematic review. Int. J. Behav. Nutr. Phys. Act..

[27] Pejtersen J.H., Kristensen T.S., Borg V., Bjorner J.B. (2010). The second version of the Copenhagen Psychosocial Questionnaire. Scand. J. Public Health.

[28] Wild D., Grove A., Martin M., Eremenco S., McElroy S., Verjee-Lorenz A., Erikson P. (2005). Principles of good practice for the translation and cultural adaptation process for patient-reported outcomes (PRO) measures: Report of the ISPOR task force for translation and cultural adaptation. Value Health.

[29] Beaton D.E., Bombardier C., Guillemin F., Ferraz M.B. (2000). Guidelines for the process of cross-cultural adaptation of self-report measures. Spine.

[30] Osler M., Heitmann B.L. (1996). The Validity of a Short Food Frequency Questionnaire and its Ability to Measure Changes in Food Intake: A Longitudinal Study. Int. J. Epidemiol..

[31] Cooper R., Al-Alami U. (2011). Food consumption patterns of female undergraduate students in the United Arab Emirates. West Afr. J. Med..

[32] Musaiger A.O., Abuirmeileh N.M. (1998). Food consumption patterns of adults in the United Arab Emirates. J. R. Soc. Promot. Health.

[33] Streiner D.L. (2003). Starting at the Beginning: An Introduction to Coefficient Alpha and Internal Consistency. J. Personal. Assess..

[34] Panagiotakos D.B., Pitsavos C., Stefanadis C. (2006). Dietary patterns: A Mediterranean diet score and its relation to clinical and biological markers of cardiovascular disease risk. Nutr. Metab. Cardiovasc. Dis..

[35] Bhutani S., Cooper J.A. (2020). COVID-19 related home confinement in adults: Weight gain risks and opportunities. Obesity.

[36] Di Renzo L., Gualtieri P., Pivari F., Soldati L., Attinà A., Cinelli G., Leggeri C., Caparello G., Barrea L., Scerbo F. (2020). Eating habits and lifestyle changes during COVID-19 lockdown: An Italian survey. J. Transl. Med..

[37] Husain W., Ashkanani F. (2020). Does COVID-19 Change Dietary Habits and Lifestyle Behaviours in Kuwait?. Environ. Health Prev. Med..

[38] ALMughamis N.S., AlAsfour S., Mehmood S. (2020). Poor Eating Habits and Predictors of Weight Gain During the COVID-19 Quarantine Measures in Kuwait: A Cross Sectional Study. Res. Sq..

[39] Moynihan A.B., Van Tilburg W.A., Igou E.R., Wisman A., Donnelly A.E., Mulcaire J.B. (2015). Eaten up by boredom: Consuming food to escape awareness of the bored self. Front. Psychol..

[40] Gardner B., Rebar A.L. (2019). Habit Formation and Behavior Change. Oxford Research Encyclopedia of Psychology.

[41] Mattioli A.V., Puviani M.B., Nasi M., Farinetti A. (2020). COVID-19 pandemic: The effects of quarantine on cardiovascular risk. Eur. J. Clin. Nutr..

[42] Sulaiman N., Elbadawi S., Hussein A., Abusnana S., Madani A., Mairghani M., Alawadi F., Sulaiman A., Zimmet P., Huse O. (2017). Prevalence of overweight and obesity in United Arab Emirates Expatriates: The UAE national diabetes and lifestyle study. Diabetol. Metab. Syndr..

[43] Razzak H.A., El-Metwally A., Harbi A., Al-Shujairi A., Qawas A. (2017). The prevalence and risk factors of obesity in the United Arab Emirates. Saudi J. Obes..

[44] Belal A.M. (2009). Nutrition-related chronic diseases Epidemic in UAE: Can we stand to STOP it?. Sudan. J. Public Health.

[45] Makansi N., Allison P., Awad M., Bedos C. (2018). Fruit and vegetable intake among Emirati adolescents: A mixed methods study. East. Mediterr. Health J..

[46] Díez J., Bilal U., Franco M. (2019). Unique features of the Mediterranean food environment: Implications for the prevention of chronic diseases Rh: Mediterranean food environments. Eur. J. Clin. Nutr..

[47] Martínez-González M.A., Gea A., Ruiz-Canela M. (2019). The Mediterranean diet and cardiovascular health: A critical review. Circ. Res..

[48] Becerra-Tomás N., Blanco Mejía S., Viguiliouk E., Khan T., Kendall C.W., Kahleova H., Rahelić D., Sievenpiper J.L., Salas-Salvadó J. (2020). Mediterranean diet, cardiovascular disease and mortality in diabetes: A systematic review and meta-analysis of prospective cohort studies and randomized clinical trials. Crit. Rev. Food Sci. Nutr..

[49] Godos J., Zappala G., Bernardini S., Giambini I., Bes-Rastrollo M., Martinez-Gonzalez M. (2017). Adherence to the Mediterranean diet is inversely associated with metabolic syndrome occurrence: A meta-analysis of observational studies. Int. J. Food Sci. Nutr..

[50] Germani A., Vitiello V., Giusti A.M., Pinto A., Donini L.M., del Balzo V. (2014). Environmental and economic sustainability of the Mediterranean Diet. Int. J. Food Sci. Nutr..

[51] Giugliano D., Esposito K. (2008). Mediterranean diet and metabolic diseases. Curr. Opin. Lipidol..

[52] Hassapidou M., Tziomalos K., Lazaridou S., Pagkalos I., Papadimitriou K., Kokkinopoulou A., Tzotzas T. (2020). The Nutrition Health Alliance (NutriHeAl) Study: A Randomized, Controlled, Nutritional Intervention Based on Mediterranean Diet in Greek Municipalities. J. Am. Coll. Nutr..

[53] Sánchez-Villegas A., Bes-Rastrollo M., Martínez-González M.A., Serra-Majem L. (2006). Adherence to a Mediterranean dietary pattern and weight gain in a follow-up study: The SUN cohort. Int. J. Obes..

[54] Serra-Majem L., Roman-Vinas B., Sanchez-Villegas A., Guasch-Ferre M., Corella D., La Vecchia C. (2019). Benefits of the Mediterranean diet: Epidemiological and molecular aspects. Mol. Asp. Med..

[55] Martínez-González M.A., Salas-Salvadó J., Estruch R., Corella D., Fitó M., Ros E. (2015). Benefits of the Mediterranean Diet: Insights From the PREDIMED Study. Prog. Cardiovasc. Dis..

[56] Turk-Adawi K., Sarrafzadegan N., Fadhil I., Taubert K., Sadeghi M., Wenger N.K., Tan N.S., Grace S.L. (2018). Cardiovascular disease in the Eastern Mediterranean region: Epidemiology and risk factor burden. Nat. Rev. Cardiol..

[57] Mahmoud I., Sulaiman N. (2019). Dyslipidaemia prevalence and associated risk factors in the United Arab Emirates: A population-based study. BMJ Open.

[58] Taha Z., Eltom S.E. (2018). The Role of Diet and Lifestyle in Women with Breast Cancer: An Update Review of Related Research in the Middle East. Biores. Open Access.

[59] Musaiger A.O., Al-Hazzaa H.M. (2012). Prevalence and risk factors associated with nutrition-related noncommunicable diseases in the Eastern Mediterranean region. Int. J. Gen. Med..

[60] Galal O. (2003). Nutrition-related health patterns in the Middle East. Asia Pac. J. Clin. Nutr..

[61] Gombart A.F., Pierre A., Maggini S. (2020). A review of micronutrients and the immune System–Working in harmony to reduce the risk of infection. Nutrients.

[62] Ipsos 5 Ways COVID-19 Has Impacted MENA’s Food Habits. https://www.ipsos.com/sites/default/files/ct/news/documents/2020-06/5_ways_covid-19_impacted_menas_food_habits_-_ipsos_mena_0.pdf.

[63] Ammar A., Brach M., Trabelsi K., Chtourou H., Boukhris O., Masmoudi L., Bouaziz B., Bentlage E., How D., Ahmed M. (2020). Effects of COVID-19 home confinement on physical activity and eating behaviour Preliminary results of the ECLB-COVID19 international online-survey. medRxiv.

[64] Abbas A.M., Fathy S.K., Fawzy A.T., Salem A.S., Shawky M.S. (2020). The mutual effects of COVID-19 and obesity. Obes. Med..

[65] Burtscher J., Burtscher M., Millet G.P. (2020). (Indoor) isolation, stress and physical inactivity: Vicious circles accelerated by Covid-19?. Scand. J. Med. Sci. Sports.

[66] Jiménez-Pavón D., Carbonell-Baeza A., Lavie C.J. (2020). Physical exercise as therapy to fight against the mental and physical consequences of COVID-19 quarantine: Special focus in older people. Prog. Cardiovasc. Dis..

[67] Czosnek L., Lederman O., Cormie P., Zopf E., Stubbs B., Rosenbaum S. (2019). Health benefits, safety and cost of physical activity interventions for mental health conditions: A meta-review to inform translation efforts. Ment. Health Phys. Act..

[68] Shigemura J., Ursano R.J., Morganstein J.C., Kurosawa M., Benedek D.M. (2020). Public responses to the novel 2019 coronavirus (2019-nCoV) in Japan: Mental health consequences and target populations. Psychiatry Clin. Neurosci..

[69] Wang C., Pan R., Wan X., Tan Y., Xu L., Ho C.S., Ho R.C. (2020). Immediate psychological responses and associated factors during the initial stage of the 2019 coronavirus disease (COVID-19) epidemic among the general population in China. Int. J. Environ. Res. Public Health.

[70] Zandifar A., Badrfam R. (2020). Iranian mental health during the COVID-19 epidemic. Asian J. Psychiatry.

[71] World Health Organization (2020). Mental Health and Psychosocial Considerations during the COVID-19 Outbreak, 18 March 2020.

[72] Cinelli M., Quattrociocchi W., Galeazzi A., Valensise C.M., Brugnoli E., Schmidt A.L., Zola P., Zollo F., Scala A. (2020). The covid-19 social media infodemic. arXiv.

[73] Gao J., Zheng P., Jia Y., Chen H., Mao Y., Chen S., Wang Y., Fu H., Dai J. (2020). Mental health problems and social media exposure during COVID-19 outbreak. PLoS ONE.

[74] Xiao H., Zhang Y., Kong D., Li S., Yang N. (2020). The effects of social support on sleep quality of medical staff treating patients with coronavirus disease 2019 (COVID-19) in January and February 2020 in China. Med. Sci. Monit. Int. Med. J. Exp. Clin. Res..

[75] Zhou X., Snoswell C.L., Harding L.E., Bambling M., Edirippulige S., Bai X., Smith A.C. (2020). The role of telehealth in reducing the mental health burden from COVID-19. Telemed. e-Health.

[76] Rosato V., Temple N.J., La Vecchia C., Castellan G., Tavani A., Guercio V. (2019). Mediterranean diet and cardiovascular disease: A systematic review and meta-analysis of observational studies. Eur. J. Nutr..

[77] Salvatore F.P., Relja A., Filipčić I.Š., Polašek O., Kolčić I. (2019). Mediterranean diet and mental distress:“10,001 Dalmatians” study. Br. Food J..

[78] Godos J., Ferri R., Caraci F., Cosentino F.I.I., Castellano S., Galvano F., Grosso G. (2019). Adherence to the mediterranean diet is associated with better sleep quality in Italian adults. Nutrients.

[79] Muñoz M.A., Fíto M., Marrugat J., Covas M.I., Schröder H. (2008). Adherence to the Mediterranean diet is associated with better mental and physical health. Br. J. Nutr..

[80] Smith G. (2008). Does Gender Influence Online Survey Participation: A Record-Linkage Analysis of University Faculty Online Survey Response Behavior.

