# Supplementary material for: Eating Habits and Lifestyle during COVID-19 Lockdown in the United Arab Emirates: A Cross-Sectional Study
Source: Nutrients. 2020 Oct 29;12(11):3314. doi: 10.3390/nu12113314 (PMC7693610; doi:10.3390/nu12113314)
Supplement: Supplementary file 1 [file nutrients-12-03314-s001.pdf]

Eating Habits and Lifestyle during COVID-19 Lockdown in the United Arab Emirates: A Cross-Sectional Study

Personal data

1. **What is your gender?**
   - Female
   - Male
2. **Which age group do you belong to?**
   - 18-25
   - 26-35
   - 36-45
   - 46-55
   - >56
3. **What is your marital status?**
   - Married
   - Single
   - Divorced
   - Widowed
4. **How many kids do you have?**
   - I have no kids
   - 1-2 kids
   - 3 or more kids
5. **What is your education Level?**
   - Less than high school
   - High School
   - College/ Diploma
   - University Degree
   - Higher education masters/doctorate
6. **What is your employment status?**
   - Full-time employment
   - Part-time employment
   - Unemployed
   - Retired
   - Self-employed
   - Student
7. **Do you currently work/study from home?**
   - Yes
   - No

- Not applicable

8. **Did your weight change during coronavirus pandemic?**
    - Lost weight
    - Gained weight
    - Maintained weight
    - I don't know
9. **What option describes your general state of health in the past 3 months?**
    - Excellent
    - Very good
    - Good
    - Fair
    - Poor
10. **Emirate of residence? ----**

Sources of information

1. **Where do you get health-related information from?** ***(choose all that apply)***
    - ○ Local and international health authorities
    - ○ Websites and social media
    - ○ Healthcare professionals
    - ○ Television
    - ○ Newspapers
    - ○ Friends and family
2. **Where do you get food and nutrition related information from?** ***(choose all that apply)***
    - Local and international health authorities
    - Websites and social media
    - Healthcare professionals
    - Television
    - Newspapers
    - Friends and family

Eating Habits

1. **Before coronavirus pandemic, most of your consumed meals during the week were?**
    - Homemade
    - Frozen ready-to-eat meals
    - Fast food (take-away, delivery)
    - Restaurants (take-away, delivery)
    - Healthy food (take-away, delivery)
2. **During coronavirus pandemic most of your consumed meals during the week are?**
    - Homemade
    - Frozen ready-to-eat meals
    - Fast food (take-away, delivery)
    - Restaurants (take-away, delivery)
    - Healthy food (take-away, delivery)
3. **How many meals did you eat per day before coronavirus pandemic?**
    - 1-2
    - 3-4
    - More than 5
4. **How many meals do you eat per day during coronavirus pandemic?**
    - 1-2
    - 3-4
    - More than 5
5. **Did you use to eat breakfast on most days of the week before coronavirus pandemic?**
    - Yes
    - No
6. **During coronavirus pandemic, do you eat breakfast on most days of the week?**
    - Yes
    - No
7. **Before coronavirus pandemic, did you use to skip meals?**
    - Yes
    - No

If yes, why is that?

- To reduce food intake
- Lack of time
- To lose weight
- Lack of appetite
- Fasting

8. **During coronavirus pandemic, are you skipping meals?**

- Yes
- No

If yes, why is that?

- To reduce food intake
- Lack of time
- To lose weight
- Lack of appetite
- Fasting

9. **Before coronavirus pandemic, how much water did you use to drink daily?**
    - 1-4 cups
    - 5-7 cups
    - 8 cups or more
10. **During coronavirus pandemic, how much water do you drink daily?**
    - 1-4 cups
    - 5-7 cups
    - 8 cups or more
11. **How often do you eat the following foods listed below?**

**Food Item**	**Never**	**1-4 times/week**	**Once/day**	**2-3 times/day**	**4 or more times/day**
Fruits					
Vegetables					
Milk and milk products					
Meat/Chicken/Fish					
Bread/rice/pasta					
Sweets/ desserts					
Coffee/Tea					
Sweet drinks (soft drinks, canned juice, etc.)					
Energy drinks					

Shopping

1. **Do you usually prepare a list before grocery shopping?**
    - Yes
    - No
2. **During coronavirus pandemic, did you start stocking up on food?**
    - Yes
    - No
    - I already stock up on food
3. **Would you rather order your groceries online (delivered to your house)?**
    - Yes
    - No
4. **Do you check food labels before purchasing?**
    - Yes
    - No
    - Sometimes
5. **Do you clean and sanitize your groceries before storage?**
    - Yes
    - No
    - Sometimes

Physical Activity

1. **Did you use to exercise before coronavirus pandemic?**
    - No
    - 1-3 times/week
    - >3 times/week
2. **During coronavirus pandemic, do you do any exercise (walking, running, equipment, etc.)?**
    - Never *(skip to Question 3)*
    - 1-3 times/week
    - >3 times/week
3. **Before coronavirus pandemic, did you use to do household chores?**
    - Never
    - 1-3 times/week
    - 4-5 times/week
    - Everyday
4. **During coronavirus pandemic, do you do household chores?**
    - Never
    - 1-3 times/week
    - 4-5 times/week
    - Everyday
5. **Before coronavirus pandemic, how much time did you spend on the computer for work/ study daily?**
    - None
    - 1-2 hours
    - 3-5 hours
    - More than 5 hours
6. **During coronavirus pandemic, how much time do you spend on the computer for work/ study daily?**
    - None
    - 1-2 hours
    - 3-5 hours
    - More than 5 hours
7. **Before coronavirus pandemic, how much time did you spend daily on Television, computer, social media for entertainment?**
    - Less than 30 minutes
    - 1-2 hours
    - 3-5 hours

- More than 5 hours

8. **During coronavirus pandemic, how much time do you spend daily on Television, computer, social media for entertainment?**
    - Less than 30 minutes
    - 1-2 hours
    - 3-5 hours
    - More than 5 hours

Stress and Irritability

1. How often have you experienced the following before coronavirus pandemic?

	All the time	A large part of the time	Part of the time	A small part of the time	Not at all
Physically exhausted					
Emotionally exhausted					
Irritable					
Tense					

2. How often have you experienced the following during coronavirus pandemic?

	All the time	A large part of the time	Part of the time	A small part of the time	Not at all
Physically exhausted					
Emotionally exhausted					
Irritable					
Tense					

Sleep

1. **Before coronavirus pandemic, how many hours did you sleep per night?**
    - Less than 7 hours
    - 7-9 hours
    - More than 9 hours
2. **During coronavirus pandemic, how many hours are you sleeping per night?**
    - Less than 7 hours
    - 7-9 hours
    - More than 9 hours
3. **How would you rate your sleep quality before coronavirus pandemic?**
    - Very good
    - Good
    - Poor
4. **How would you rate your sleep quality during coronavirus pandemic?**
    - Very good
    - Good
    - Poor

5. **Before coronavirus pandemic, did you experience any of the following? (choose all that apply)**
    - Slept badly and restlessly
    - Hard to go to sleep
    - Woken up too early and not been able to get back to sleep
    - Woken up several times and found it difficult to get back to sleep
    - None of the above
6. **During coronavirus pandemic, do you experience any of the following? (choose all that apply)**
    - Slept badly and restlessly
    - Hard to go to sleep
    - Woken up too early and not been able to get back to sleep
    - Woken up several times and found it difficult to get back to sleep
    - None of the above
7. **How would you describe your energy level before coronavirus pandemic?**
    - Energized
    - Neutral
    - Lazy
8. **How would you describe your energy level during coronavirus pandemic?**
    - Energized
    - Neutral
    - Lazy
